# Poly[bis­[μ-1,4-bis­(imidazol-1-ylmeth­yl)benzene]dichloridocadmium(II)]

**DOI:** 10.1107/S1600536808018990

**Published:** 2008-06-28

**Authors:** Yu Ding, Genwen Zheng, Zhengbing Fu, Xinliang Hu

**Affiliations:** a Department of Chemistry, Xiaogan University, Xiaogan, Hubei 432000, People’s Republic of China

## Abstract

The title compound, [CdCl_2_(C_14_H_14_N_4_)_2_]_*n*_, has a slightly distorted octa­hedral coordination geometry, formed by four N atoms from 1,4-bis­(imidazol-1-ylmeth­yl)benzene ligands and two Cl atoms, giving a two-dimensional network. The Cd atom lies on a centre of inversion.

## Related literature

For related literature, see: Zhou & Du (2007 ▶). 
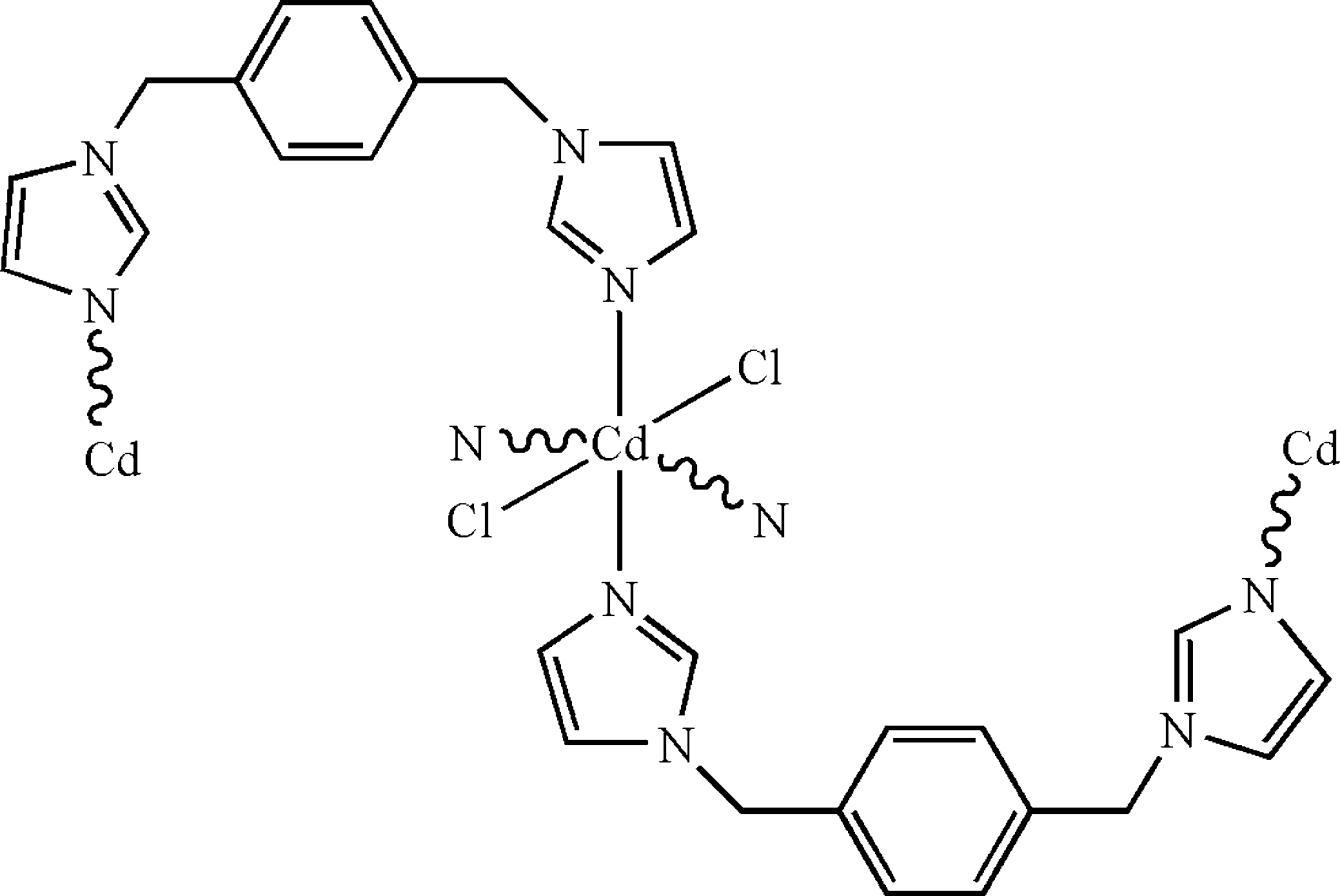


## Experimental

### 

#### Crystal data

[CdCl_2_(C_14_H_14_N_4_)_2_]
*M* *_r_* = 659.88
Monoclinic, 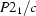

*a* = 7.7983 (3) Å
*b* = 12.8274 (6) Å
*c* = 14.4190 (6) Å
β = 105.642 (1)°
*V* = 1388.94 (10) Å^3^
*Z* = 2
Mo *K*α radiation
μ = 1.01 mm^−1^
*T* = 293 (2) K
0.32 × 0.26 × 0.24 mm

#### Data collection

Bruker SMART APEX CCD area-detector diffractometer
Absorption correction: multi-scan (*SAINT*; Bruker, 2001 ▶) *T*_min_ = 0.74, *T*_max_ = 0.78
7453 measured reflections
2728 independent reflections
2331 reflections with *I* > 2σ(*I*)
*R*_int_ = 0.037

#### Refinement

*R*[*F* ^2^ > 2σ(*F* ^2^)] = 0.024
*wR*(*F* ^2^) = 0.058
*S* = 1.00
2728 reflections
178 parameters
H-atom parameters constrained
Δρ_max_ = 0.34 e Å^−3^
Δρ_min_ = −0.43 e Å^−3^

### 

Data collection: *SMART* (Bruker, 2001 ▶); cell refinement: *SAINT* (Bruker, 2001 ▶); data reduction: *SAINT*; program(s) used to solve structure: *SHELXTL* (Sheldrick, 2008 ▶); program(s) used to refine structure: *SHELXTL*; molecular graphics: *SHELXTL*; software used to prepare material for publication: *SHELXTL*.

## Supplementary Material

Crystal structure: contains datablocks I, global. DOI: 10.1107/S1600536808018990/sg2246sup1.cif

Structure factors: contains datablocks I. DOI: 10.1107/S1600536808018990/sg2246Isup2.hkl

Additional supplementary materials:  crystallographic information; 3D view; checkCIF report

## Figures and Tables

**Table:** 

Cd1—N1^i^	2.3546 (17)
Cd1—N1	2.3546 (17)
Cd1—N3	2.3561 (15)
Cd1—N3^i^	2.3561 (15)
Cd1—Cl1^i^	2.6248 (5)
Cd1—Cl1	2.6248 (5)

**Table:** 

N1^i^—Cd1—N1	180
N1^i^—Cd1—N3	94.57 (6)
N1—Cd1—N3	85.43 (6)
N3—Cd1—N3^i^	180
N1—Cd1—Cl1^i^	89.96 (4)
N1—Cd1—Cl1	90.04 (4)
N3—Cd1—Cl1	89.60 (4)
N3^i^—Cd1—Cl1	90.40 (4)
Cl1^i^—Cd1—Cl1	180

Symmetry code: (i) 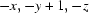.

**Fig. 1.**
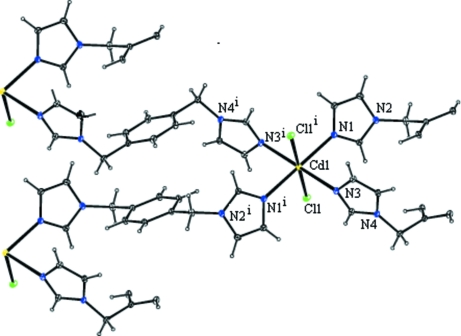
The molecular structure of (I), showing ellipsoids at the 30% probability level. H atoms have been omitted for clarity. [Symmetry codes: (i)-x,-y + 1,-z]

**Fig. 2.**
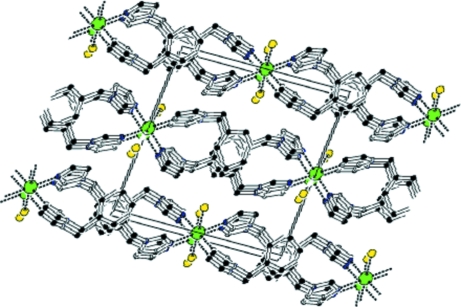
The molecular packing diagram of (I), with hydrogen bonds shown as dashed lines.

## supplementary crystallographic information

### Comment

The self-assembly reactions of M^II^Cl_2_ (M = Ni, Co) with the flexible bix ligand with 2D network [Ni(bix)_2_Cl_2_]_n_ and a 1D chain [Co(bix)Cl_2_]_n_ had been reported (Zhou *et al.*, 2007). Here we report a new polymer. The Cd(II) center is coordinated by four nitrogen atoms from four bix ligands and two Cl atoms to furnish a slightly distorted octahedral. Four nitrogen atoms (N1, N1^i^, N3, and N3^i^) [symmetry code: (i) -*x*,-*y* + 1,-*z*] occupy the equatorial positions. The axial positions are occupied by two Cl donors. Each Cd(II) ion is linked by four µ2-birdging bix ligands to its four neighboring Cd(II) ions, thus affording two-dimensional grid layers (Fig. 2) in the direction of *b* axis. There are two different types of *µ*^2^-bridging bix ligands in the compound.

The ring centriods distances and displacement angles of of *Cg*1···*Cg*1^i^ is 1.573 Å and 0.03° [*Cg*1 is N1—C1 ring centroid; symmetry code: (i) -*x*,-*y* + 1,-*z*], suggesting significant π-π interactions. which are the factors that stabilized the crystal structure.

### Experimental

Compound (I) was was prepared as follows: to a solution of bix dihydrate 0.14 g(0.5 mmol) and water (8 mL) was added CdCl_2_ (0.092 g, 0.5 mmol). The mixture was sealed in a stainless steel reactor with a Teflon liner, and was heated to 393 K for two days. After slow cooling to room temperature, colourless block-shaped crystals of (I) were obtained.

### Refinement

The H atoms bonded to C atoms were introduced at calculated positions and refined using a riding model, with *U*_iso_(H) = 1.2Ueq(C) and *U*_iso_(H) = 1.5Ueq(C), and C–H distances of 0.93–0.96 Å.

### Crystal data

**Table:**

[CdCl_2_(C_14_H_14_N_4_)_2_]	*F*_000_ = 668
*M**_r_* = 659.88	*D*_x_ = 1.578 Mg m^−^^3^
Monoclinic, *P*2_1_/*c*	Mo *K*α radiation λ = 0.71073 Å
Hall symbol: -P 2ybc	Cell parameters from 4013 reflections
*a* = 7.7983 (3) Å	θ = 2.7–28.3º
*b* = 12.8274 (6) Å	µ = 1.01 mm^−^^1^
*c* = 14.4190 (6) Å	*T* = 293 (2) K
β = 105.6420 (10)º	Block, colourless
*V* = 1388.94 (10) Å^3^	0.32 × 0.26 × 0.24 mm
*Z* = 2	

### Data collection

**Table:**

Bruker SMART APEX CCD area-detector diffractometer	2728 independent reflections
Radiation source: sealed tube	2331 reflections with *I* > 2σ(*I*)
Monochromator: graphite	*R*_int_ = 0.037
*T* = 293(2) K	θ_max_ = 26.0º
φ and ω scans	θ_min_ = 2.2º
Absorption correction: multi-scan(SAINT; Bruker, 2001)	*h* = −9→6
*T*_min_ = 0.74, *T*_max_ = 0.78	*k* = −15→14
7453 measured reflections	*l* = −17→17

### Refinement

**Table:**

Refinement on *F*^2^	Secondary atom site location: difference Fourier map
Least-squares matrix: full	Hydrogen site location: inferred from neighbouring sites
*R*[*F*^2^ > 2σ(*F*^2^)] = 0.024	H-atom parameters constrained
*wR*(*F*^2^) = 0.058	*w* = 1/[σ^2^(*F*_o_^2^) + (0.0298*P*)^2^] where *P* = (*F*_o_^2^ + 2*F*_c_^2^)/3
*S* = 1.01	(Δ/σ)_max_ = 0.001
2728 reflections	Δρ_max_ = 0.34 e Å^−^^3^
178 parameters	Δρ_min_ = −0.43 e Å^−^^3^
Primary atom site location: structure-invariant direct methods	Extinction correction: none

### Special details

**Table:**

Geometry. All e.s.d.'s (except the e.s.d. in the dihedral angle between two l.s. planes) are estimated using the full covariance matrix. The cell e.s.d.'s are taken into account individually in the estimation of e.s.d.'s in distances, angles and torsion angles; correlations between e.s.d.'s in cell parameters are only used when they are defined by crystal symmetry. An approximate (isotropic) treatment of cell e.s.d.'s is used for estimating e.s.d.'s involving l.s. planes.
Refinement. Refinement of *F*^2^ against ALL reflections. The weighted *R*-factor *wR* and goodness of fit *S* are based on *F*^2^, conventional *R*-factors *R* are based on *F*, with *F* set to zero for negative *F*^2^. The threshold expression of *F*^2^ > σ(*F*^2^) is used only for calculating *R*-factors(gt) *etc*. and is not relevant to the choice of reflections for refinement. *R*-factors based on *F*^2^ are statistically about twice as large as those based on *F*, and *R*- factors based on ALL data will be even larger.

### Fractional atomic coordinates and isotropic or equivalent isotropic displacement parameters (Å^2^)

**Table:**

	*x*	*y*	*z*	*U*_iso_*/*U*_eq_	
Cd1	0.0000	0.5000	0.0000	0.02725 (8)	
C1	0.3962 (3)	0.60574 (17)	0.12383 (16)	0.0408 (5)	
H1	0.4470	0.5971	0.0729	0.049*	
C2	0.2078 (3)	0.60595 (15)	0.20672 (14)	0.0323 (5)	
H2	0.1033	0.5975	0.2253	0.039*	
C3	0.4819 (3)	0.64371 (17)	0.21155 (16)	0.0418 (5)	
H3	0.5999	0.6653	0.2321	0.050*	
C4	0.3888 (3)	0.67235 (16)	0.36600 (15)	0.0411 (5)	
H4A	0.2794	0.7003	0.3757	0.049*	
H4B	0.4784	0.7268	0.3818	0.049*	
C5	0.4493 (3)	0.58124 (15)	0.43373 (14)	0.0300 (4)	
C6	0.3967 (3)	0.47986 (15)	0.40840 (16)	0.0367 (5)	
H6	0.3268	0.4656	0.3465	0.044*	
C7	0.4469 (3)	0.39939 (16)	0.47405 (15)	0.0353 (5)	
H7	0.4103	0.3317	0.4559	0.042*	
C8	0.0724 (3)	0.33407 (15)	0.18324 (15)	0.0351 (5)	
H8	0.1700	0.3061	0.1665	0.042*	
C9	0.0320 (3)	0.31662 (16)	0.26739 (15)	0.0361 (5)	
H9	0.0957	0.2760	0.3186	0.043*	
C10	−0.1663 (3)	0.41959 (15)	0.17628 (14)	0.0340 (5)	
H10	−0.2654	0.4624	0.1550	0.041*	
C11	−0.2121 (3)	0.38072 (18)	0.33871 (15)	0.0434 (6)	
H11A	−0.2346	0.3118	0.3606	0.052*	
H11B	−0.3260	0.4149	0.3132	0.052*	
C12	−0.1024 (3)	0.44253 (16)	0.42282 (14)	0.0338 (5)	
C13	−0.0831 (3)	0.54885 (18)	0.41550 (15)	0.0413 (5)	
H13	−0.1394	0.5825	0.3582	0.050*	
C14	−0.0181 (3)	0.39392 (18)	0.50832 (14)	0.0401 (5)	
H14	−0.0296	0.3223	0.5146	0.048*	
Cl1	−0.23305 (7)	0.63726 (4)	0.02514 (4)	0.04181 (14)	
N1	0.2237 (2)	0.58192 (13)	0.12087 (12)	0.0331 (4)	
N2	0.3585 (2)	0.64392 (12)	0.26426 (12)	0.0330 (4)	
N3	−0.0517 (2)	0.39902 (12)	0.12627 (12)	0.0338 (4)	
N4	−0.1214 (2)	0.37075 (13)	0.26207 (11)	0.0327 (4)	

### Atomic displacement parameters (Å^2^)

**Table:**

	*U*^11^	*U*^22^	*U*^33^	*U*^12^	*U*^13^	*U*^23^
Cd1	0.03452 (13)	0.02730 (12)	0.02127 (12)	0.00197 (8)	0.00982 (9)	0.00096 (8)
C1	0.0377 (13)	0.0508 (13)	0.0369 (12)	0.0034 (11)	0.0151 (10)	0.0020 (10)
C2	0.0337 (12)	0.0325 (11)	0.0313 (11)	0.0002 (9)	0.0098 (9)	0.0016 (9)
C3	0.0301 (12)	0.0469 (13)	0.0461 (14)	−0.0008 (10)	0.0066 (11)	0.0077 (11)
C4	0.0568 (15)	0.0319 (11)	0.0292 (11)	−0.0001 (10)	0.0025 (11)	−0.0002 (9)
C5	0.0343 (11)	0.0285 (10)	0.0257 (10)	−0.0028 (8)	0.0058 (9)	0.0005 (8)
C6	0.0433 (13)	0.0334 (12)	0.0261 (11)	−0.0056 (9)	−0.0030 (10)	−0.0027 (9)
C7	0.0459 (13)	0.0266 (11)	0.0292 (10)	−0.0076 (9)	0.0029 (9)	−0.0031 (9)
C8	0.0409 (13)	0.0314 (11)	0.0353 (11)	0.0025 (9)	0.0140 (10)	0.0016 (9)
C9	0.0439 (13)	0.0341 (11)	0.0279 (11)	−0.0023 (10)	0.0057 (10)	0.0046 (9)
C10	0.0409 (12)	0.0328 (11)	0.0295 (11)	−0.0005 (9)	0.0120 (10)	−0.0002 (9)
C11	0.0512 (14)	0.0534 (14)	0.0322 (12)	−0.0177 (11)	0.0226 (11)	−0.0107 (10)
C12	0.0410 (13)	0.0368 (12)	0.0281 (11)	−0.0056 (10)	0.0171 (10)	−0.0057 (9)
C13	0.0535 (15)	0.0399 (13)	0.0289 (11)	0.0021 (11)	0.0081 (11)	0.0056 (10)
C14	0.0607 (16)	0.0270 (11)	0.0348 (12)	−0.0030 (10)	0.0169 (11)	0.0005 (9)
Cl1	0.0455 (3)	0.0355 (3)	0.0504 (3)	0.0090 (2)	0.0233 (3)	−0.0009 (2)
N1	0.0341 (10)	0.0366 (10)	0.0293 (9)	0.0022 (8)	0.0098 (8)	−0.0002 (7)
N2	0.0393 (10)	0.0302 (9)	0.0265 (9)	−0.0012 (8)	0.0035 (8)	0.0031 (7)
N3	0.0437 (11)	0.0325 (9)	0.0276 (9)	0.0018 (8)	0.0139 (8)	0.0038 (7)
N4	0.0427 (11)	0.0347 (9)	0.0235 (9)	−0.0087 (8)	0.0139 (8)	−0.0030 (7)

### Geometric parameters (Å, °)

**Table:**

Cd1—N1^i^	2.3546 (17)	C6—H6	0.9300
Cd1—N1	2.3546 (17)	C7—C5^ii^	1.380 (3)
Cd1—N3	2.3561 (15)	C7—H7	0.9300
Cd1—N3^i^	2.3561 (15)	C8—C9	1.352 (3)
Cd1—Cl1^i^	2.6248 (5)	C8—N3	1.370 (3)
Cd1—Cl1	2.6248 (5)	C8—H8	0.9300
C1—C3	1.352 (3)	C9—N4	1.367 (3)
C1—N1	1.369 (3)	C9—H9	0.9300
C1—H1	0.9300	C10—N3	1.317 (2)
C2—N1	1.314 (2)	C10—N4	1.346 (2)
C2—N2	1.335 (3)	C10—H10	0.9300
C2—H2	0.9300	C11—N4	1.469 (2)
C3—N2	1.378 (3)	C11—C12	1.507 (3)
C3—H3	0.9300	C11—H11A	0.9700
C4—N2	1.468 (3)	C11—H11B	0.9700
C4—C5	1.514 (3)	C12—C13	1.379 (3)
C4—H4A	0.9700	C12—C14	1.380 (3)
C4—H4B	0.9700	C13—C14^iii^	1.379 (3)
C5—C7^ii^	1.380 (3)	C13—H13	0.9300
C5—C6	1.383 (3)	C14—C13^iii^	1.379 (3)
C6—C7	1.384 (3)	C14—H14	0.9300
			
N1^i^—Cd1—N1	180.00 (7)	C6—C7—H7	119.7
N1^i^—Cd1—N3	94.57 (6)	C9—C8—N3	109.92 (19)
N1—Cd1—N3	85.43 (6)	C9—C8—H8	125.0
N1^i^—Cd1—N3^i^	85.43 (6)	N3—C8—H8	125.0
N1—Cd1—N3^i^	94.57 (6)	C8—C9—N4	106.13 (18)
N3—Cd1—N3^i^	180.00 (6)	C8—C9—H9	126.9
N1^i^—Cd1—Cl1^i^	90.04 (4)	N4—C9—H9	126.9
N1—Cd1—Cl1^i^	89.96 (4)	N3—C10—N4	111.30 (19)
N3—Cd1—Cl1^i^	90.40 (4)	N3—C10—H10	124.3
N3^i^—Cd1—Cl1^i^	89.60 (4)	N4—C10—H10	124.3
N1^i^—Cd1—Cl1	89.96 (4)	N4—C11—C12	111.53 (17)
N1—Cd1—Cl1	90.04 (4)	N4—C11—H11A	109.3
N3—Cd1—Cl1	89.60 (4)	C12—C11—H11A	109.3
N3^i^—Cd1—Cl1	90.40 (4)	N4—C11—H11B	109.3
Cl1^i^—Cd1—Cl1	180.00 (2)	C12—C11—H11B	109.3
C3—C1—N1	110.09 (19)	H11A—C11—H11B	108.0
C3—C1—H1	125.0	C13—C12—C14	118.68 (18)
N1—C1—H1	125.0	C13—C12—C11	120.56 (19)
N1—C2—N2	112.35 (18)	C14—C12—C11	120.8 (2)
N1—C2—H2	123.8	C14^iii^—C13—C12	121.08 (19)
N2—C2—H2	123.8	C14^iii^—C13—H13	119.5
C1—C3—N2	105.90 (19)	C12—C13—H13	119.5
C1—C3—H3	127.0	C13^iii^—C14—C12	120.2 (2)
N2—C3—H3	127.0	C13^iii^—C14—H14	119.9
N2—C4—C5	113.02 (17)	C12—C14—H14	119.9
N2—C4—H4A	109.0	C2—N1—C1	105.06 (18)
C5—C4—H4A	109.0	C2—N1—Cd1	124.26 (14)
N2—C4—H4B	109.0	C1—N1—Cd1	130.41 (14)
C5—C4—H4B	109.0	C2—N2—C3	106.59 (17)
H4A—C4—H4B	107.8	C2—N2—C4	126.14 (18)
C7^ii^—C5—C6	118.61 (17)	C3—N2—C4	127.12 (19)
C7^ii^—C5—C4	118.82 (18)	C10—N3—C8	105.51 (16)
C6—C5—C4	122.48 (19)	C10—N3—Cd1	126.54 (13)
C5—C6—C7	120.8 (2)	C8—N3—Cd1	123.87 (13)
C5—C6—H6	119.6	C10—N4—C9	107.12 (16)
C7—C6—H6	119.6	C10—N4—C11	126.32 (19)
C5^ii^—C7—C6	120.60 (19)	C9—N4—C11	126.38 (18)
C5^ii^—C7—H7	119.7		

Symmetry codes: (i) −*x*, −*y*+1, −*z*; (ii) −*x*+1, −*y*+1, −*z*+1; (iii) −*x*, −*y*+1, −*z*+1.
